# Stereoselective transition metal-catalyzed [(2+2)+1] and [(2+2)+2] carbocyclization reactions using 1,6-enynes with 1,1-disubstituted olefins: construction of quaternary centers

**DOI:** 10.1039/d4sc02645d

**Published:** 2024-09-12

**Authors:** Ridge Michael P. Ylagan, Yu Zhu, P. Andrew Evans

**Affiliations:** a Department of Chemistry, Queen's University 90 Bader Lane Kingston Ontario K7L 3N6 Canada Andrew.Evans@chem.queensu.ca; b Xiangya School of Pharmaceutical Sciences, Central South University Changsha 410013 Hunan China

## Abstract

Transition metal-catalyzed carbocyclization reactions provide a powerful method for the stereoselective assembly of complex, highly substituted (poly)cyclic scaffolds. Although 1,6-enynes are common substrates for these transformations, using polysubstituted alkene derivatives to construct functionalized cyclic products remains challenging due to their significantly lower reactivity. This *Perspective* highlights key developments in stereoselective semi-intramolecular metal-catalyzed [(2+2)+1] and [(2+2)+2] carbocyclizations of 1,6-enynes containing 1,1-disubstituted alkenes, which produce cycloadducts with quaternary stereogenic centers. The insights gleaned from these examples provide a blueprint for developing more general carbocyclization strategies with challenging polysubstituted olefins.

## Abstract 2

The development of transition metal-catalyzed [(2+2)+1] and [(2+2)+2] carbocyclization reactions of 1,6-enynes bearing 1,1-disubstituted olefins is discussed, highlighting both the challenges and opportunities for expanding this powerful transformation.

## Graphical abstract

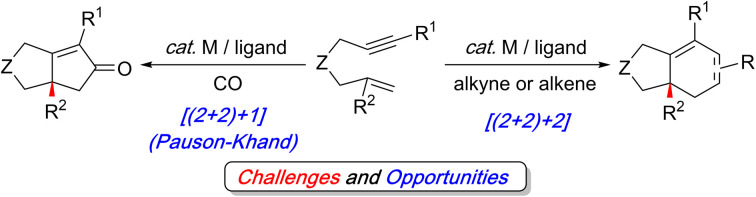

## 1. Introduction

Transition metal-catalyzed higher-order [m+n+o] carbocyclization reactions involving multiple unsaturated components (m, n, o, *etc.*), offer a powerful approach for the modular and stereoselective construction of complex and highly substituted (poly)cyclic scaffolds found in important synthetic targets.^1^ A key feature that distinguishes metal-catalyzed carbocyclizations from more conventional thermal or photochemical cycloadditions is the involvement of one or more carbometallation steps in the cyclization process.^2^ Since the seminal work of Reppe and Schweckendiek on the nickel-catalyzed [2+2+2] in 1948,^3^ this class of transformations has undergone extensive development, including the introduction of diverse metal catalysts and π-components with various substituents. As a result, this approach enables the construction of a wide array of carbo- and heterocyclic products, varying in ring size, substitution, and degree of unsaturation.

The importance of metal-catalyzed carbocyclization reactions can be attributed, at least in part, to the flexibility in tailoring the properties of the transition metal complex. For instance, the ability to systematically tune the stereoelectronic properties of the catalyst using ancillary ligands, including chiral derivatives, in conjunction with the type of metal center, often leads to the desired reactivity and selectivity.^1,2^ Additionally, the *in situ* modification of pre-catalysts to form active chiral metal complexes enhances modularity, enabling their application across diverse substrates. The carbometallation step, often both rate- and stereodetermining, is central to these transformations, facilitating the efficient formation of multiple C–C bonds in a single, atom-economical operation. This efficiency and precision have made stereoselective carbocyclizations an indispensable tool for the synthesis of polycyclic products, as demonstrated by their extensive use in the total synthesis of complex natural products. By uniting modularity, practicality, and stereochemical control, stereoselective carbocyclization reactions continue to advance synthetic methodology, offering unparalleled opportunities for constructing intricate molecular targets.

**Scheme 1:**
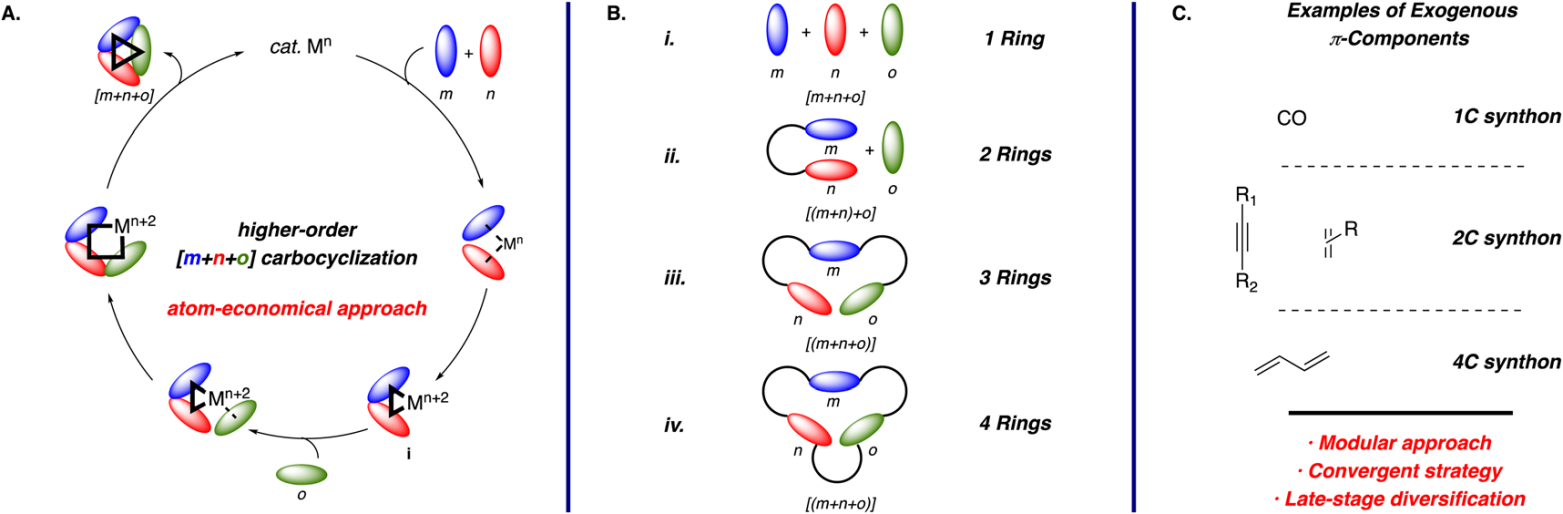
(A) General catalytic cycle for transition metal-catalyzed higher-order [m+n+o] carbocyclization reactions; (B) different reaction manifolds; and (C) selected examples of exogenous π-components employed.

The general mechanism for transition metal-catalyzed higher-order [m+n+o] carbocyclization reactions is illustrated in Scheme 1A. The catalytic cycle begins with the coordination of unsaturated components *m* and *n*, which undergo oxidative addition to form a metallacycle intermediate i. As noted above, this step is often rate- and stereodetermining, representing a critical point for catalytic control. Insertion of the third unsaturated component *o* into the metallacycle and subsequent reductive elimination forges the desired carbocycle.^1*a*^ This approach enables easy product diversification by varying the unsaturated components, showcasing the strategy's modularity. Although four-component reaction variants, such as [m+n+o+p], have been reported, they are relatively rare due to the challenges in controlling the chemoselective carbometallation steps and avoiding premature reductive elimination before the insertion of the fourth component.^1*b*^ Hence, this review focuses solely on the three-component process, *i.e.*, [m+n+o] reactions using 1,6-enynes containing 1,1-disubstituted alkenes.

Carbocyclization reactions offer significant synthetic advantages, enabling the flexible preparation of diverse (poly)cyclic scaffolds (Scheme 1B) from multiple synthons (Scheme 1C) to access a broad array of carbo- and heterocyclic products. However, addressing several critical challenges remains essential for advancing these transformations. For instance, controlling chemo- and regioselectivity in the fully intermolecular reaction (Scheme 1B(i)) is challenging and often depends on the steric^4^ or electronic^5^ bias within the substrate and the specific nature of the catalyst. In contrast, tethering two components (*m* and *n*) forms the desired metallacycle with excellent selectivity (Scheme 1B(ii)), albeit incorporating the third component (*o*) requires precise ligand control.^6^ Although the tethering of two π-components leads to the formation of an additional ring, this approach is particularly useful due to the prevalence of bicyclic structures in many synthetic targets. Hence, the semi-intermolecular reaction is one of the most popular approaches among the possible carbocyclization reaction manifolds. Finally, tethering all the unsaturated components (Scheme 1B(iii and iv)) generally provides excellent chemo- and regiocontrol, though it often requires the preparation of more elaborate substrates using multi-step sequences, which presumably explains why this approach is less commonly explored.^1,2^

**Scheme 2:**
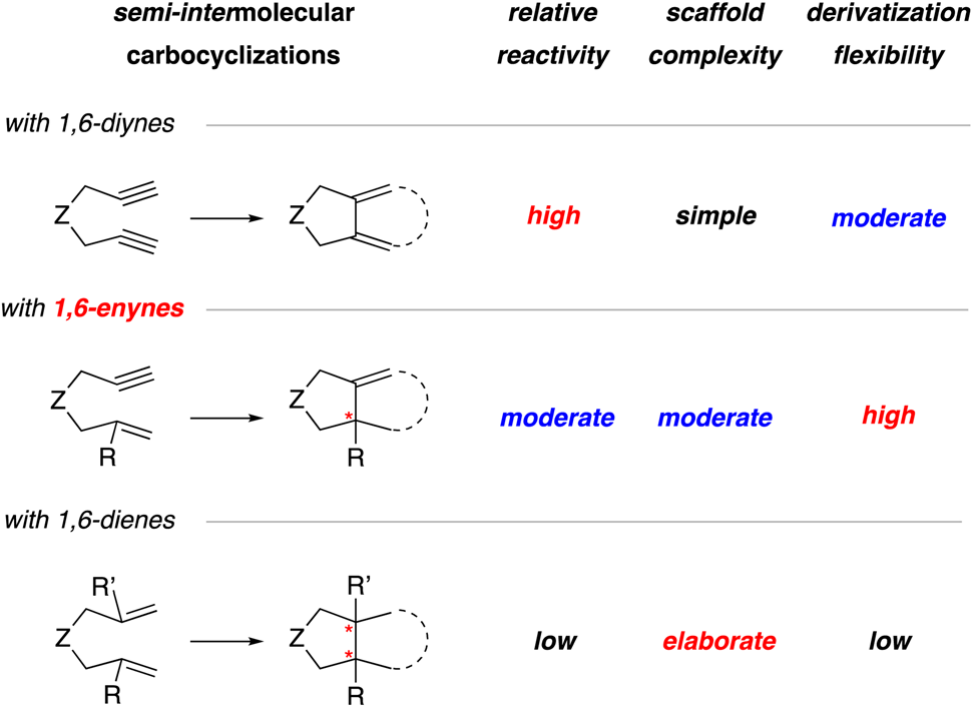
Comparison of 1,6-diynes, 1,6-enynes, and 1,6-dienes in the context of their relative reactivity in carbocyclizations, scaffold complexity, and flexibility for further functionalization.

In general, the three most common substrates used for the semi-intermolecular reaction manifold are 1,6-diynes, 1,6-enynes, and 1,6-dienes.^1,2^ This review will focus on generating stereogenic centers at the ring junction using 1,6-enynes and 1,6-dienes (Scheme 2), as 1,6-diynes typically yield either bicyclic dienes or aromatic rings. Although employing 1,6-enynes and 1,6-dienes can generate one or more stereogenic centers at the ring fusion, installing quaternary stereocenters remains particularly challenging.^7^ For instance, low reactivity is often associated with highly substituted alkenes, such as 1,1-disubstituted alkenes. Nevertheless, substituted alkenes with defined olefin geometry can, in principle, be stereospecifically converted to additional stereocenters and thereby provide an attractive strategy for target synthesis. Although 1,6-dienes have been studied, they are often limited to activated derivatives, such as 1,6-diene-enes or 1,6-allene-enes,^8,9^ to overcome the significant energy barrier for metallacycle formation.^10^ Consequently, while they can generate two stereogenic centers at the ring fusion, this reliance on activated alkenes^11^ restricts their synthetic utility. Given these considerations, 1,6-enynes are generally regarded as the optimal substrates due to their inherent reactivity, ease of assembly, broad substrate scope, and suitability for post-cyclization derivatization.

**Scheme 3:**
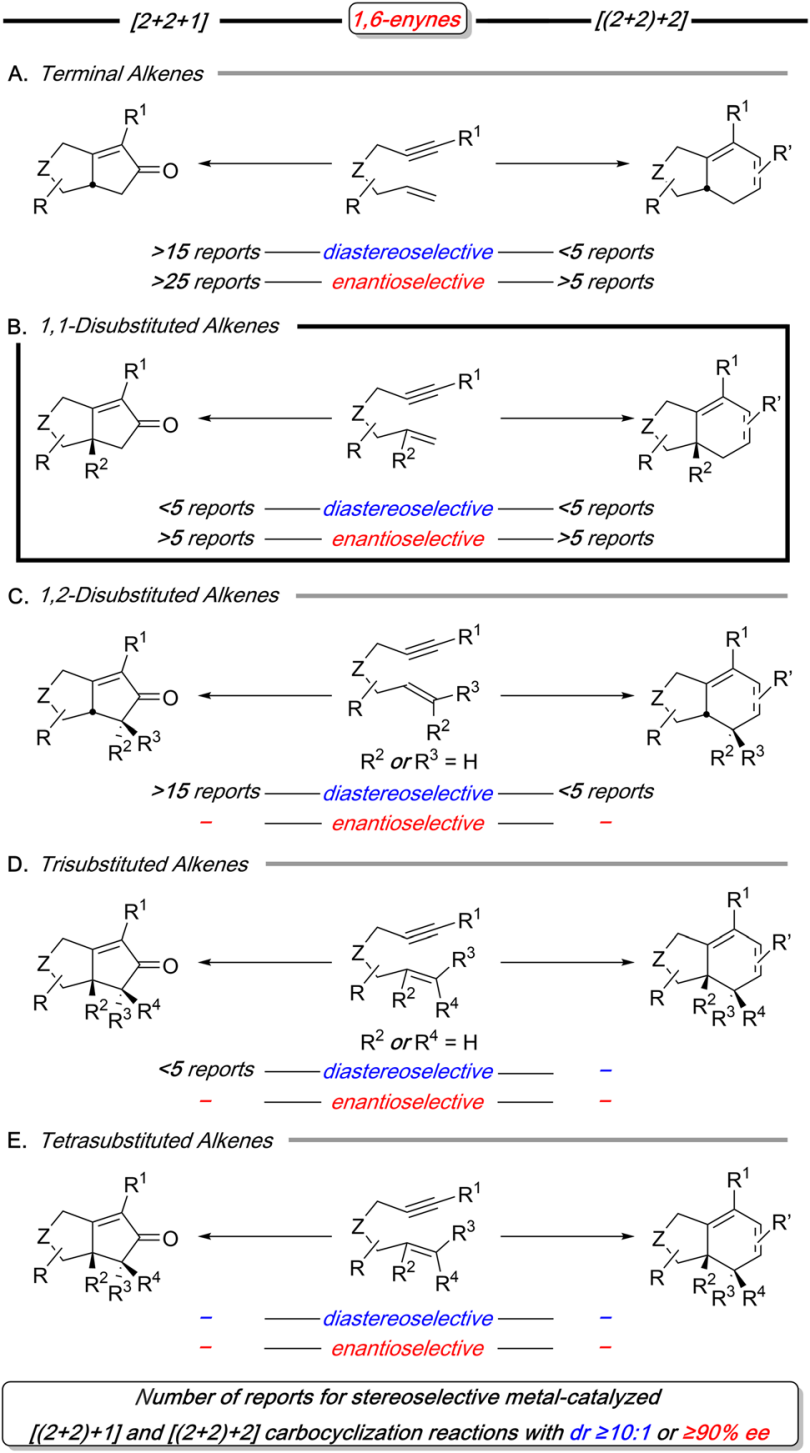
A summary of the number of reports for the stereoselective metal-catalyzed PKR (*i.e.*, [(2+2)+1]) with 1,6-enynes with increasing alkene substitution for examples with *dr* ≥10 : 1 or ≥90% *ee* (left); likewise, a summary for the stereoselective metal-catalyzed [(2+2)+2] with 1,6-enynes and alkynes/alkenes as exogenous components (right).

As a result of these considerations, 1,6-enynes have been extensively utilized in carbocyclization reactions, as exemplified by the Pauson–Khand reaction (PKR) first reported by Schore and Croudace in 1981.^12^ Although 1,6-enynes with substituted and stereodefined alkenes can produce products with one or more stereocenters, they often exhibit lower reactivity and reduced stereoselectivity. This issue is highlighted in this focused survey of 1,6-enynes used in stereoselective metal-catalyzed [(2+2)+1] reactions (PKR)^13–15^ and [(2+2)+2] carbocyclization reactions^16–18^ with alkynes and alkenes as the exogenous π-components (Scheme 3).

Our analysis of stereoselective metal-catalyzed [(2+2)+1] and [(2+2)+2] carbocyclization reactions reveal distinct trends in both diastereo- and enantioselective processes (Scheme 3). Diastereoselective reactions predominantly focus on terminal and 1,2-disubstituted alkenes (3A and 3C), with particular emphasis on the PKR reaction. However, examples featuring 1,1-disubstituted alkenes (3B) are notably limited for both transformations. Moreover, the reactions involving higher substituted olefins, such as trisubstituted and tetrasubstituted alkenes, are either rare or unknown (3D and 3E). Similarly, enantioselective reactions primarily target the terminal and 1,1-disubstituted alkenes (3A and 3B), once again prioritizing the PKR reaction. In a similar manner to the diastereoselective processes, higher substituted alkenes—such as 1,2-disubstituted, trisubstituted, and tetrasubstituted variants (3C, 3D, and 3E)—remain underrepresented for enantioselective variants. This analysis highlights the current limitations in these reactions, particularly with respect to more highly substituted alkenes. Addressing these gaps could significantly broaden the utility of 1,6-enynes in carbocyclization reactions, further enhancing their potential in synthetic chemistry.

**Scheme 4:**
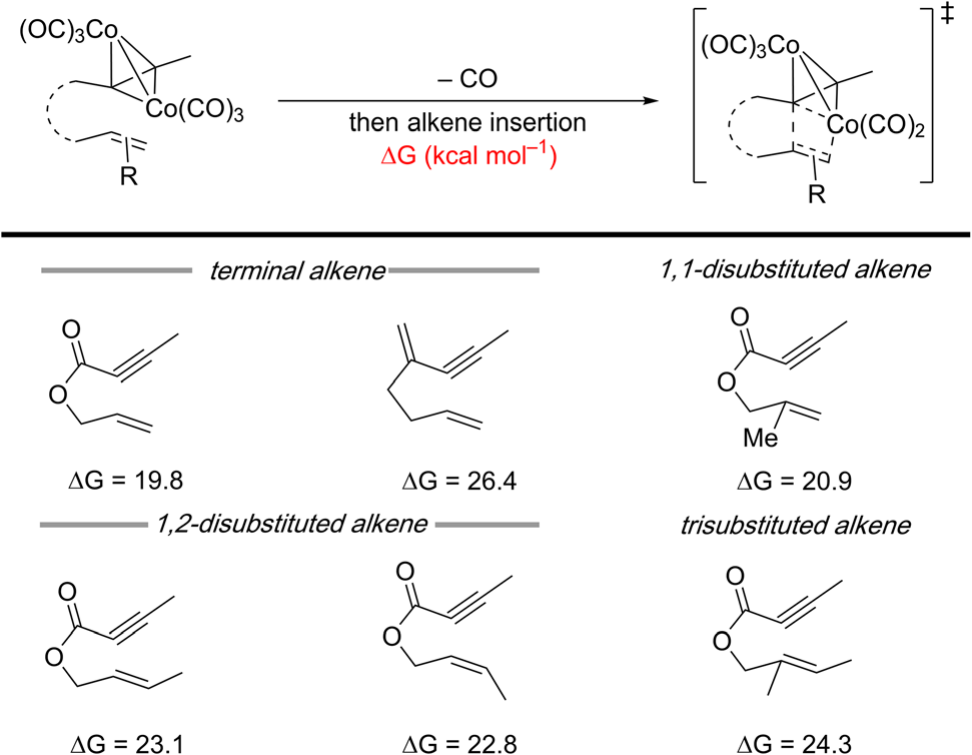
Impact of alkene substituents in 1,6-enynes on the calculated energy barrier for the alkene insertion step for cobalt-mediated PKRs.

Insight into the challenges associated with substituted olefins can be obtained from a recent computational study on a cobalt-mediated PKR using 1,6-enynes. This study demonstrates that the barrier for metallacycle formation increases with higher alkene substitution (Scheme 4).^19^ Although this is a metal-mediated computational study, its findings are still relevant for 1,6-enynes with substituted alkenes in metal-catalyzed reactions. A similar challenge likely arises with metal-catalyzed [(2+2)+2] carbocyclization reactions, as evidenced by the limited reports successfully employing 1,6-enynes with (poly)substituted alkenes (Scheme 3C–E – right arrows).

**Scheme 5:**
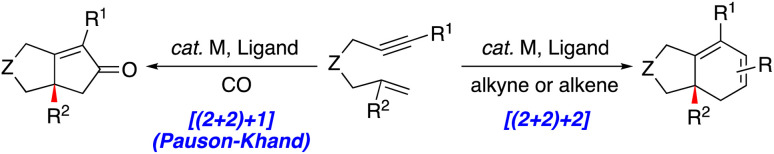
Overview of carbocyclization reactions covered in this review.

This *Perspective* summarizes the seminal studies on the transition metal-catalyzed [(2+2)+1] and [(2+2)+2] carbocyclization reactions of 1,6-enynes containing a 1,1-disubstituted alkene with either a one-carbon (1C) or two-carbon (2C) synthon (Scheme 5). The cycloaddition reactions of 1,6-enynes with other substituted alkenes, such as allenes and dienes, are outside the scope of this review,^13,16^ which focuses on the stereoselective construction of ring-fusion quaternary centers that have proven challenging (*vide supra*). These demanding substrates are expected to inspire the design and development of new variants incorporating more substituted olefins, ultimately broadening the scope of these important transformations.

## 2. Metal-catalyzed [(2+2)+1] carbocyclization reaction (PKR)

**Scheme 6:**
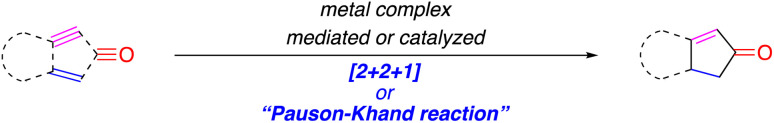
A schematic depiction of a general [2+2+1] carbocyclization reaction (PKR) with an alkyne, alkene and carbon monoxide.

The stereoselective preparation of cyclopentenones is of significant synthetic importance because it provides a scaffold present in several natural products and bioactive compounds.^20^ For instance, the discovery of prostaglandins^21^ inspired the development of several transformations for the stereoselective preparation of cyclopentenones, such as the Nazarov reaction.^22^ In contrast, the PKR permits the stereoselective construction of cyclopentenones *via* a metal-catalyzed [2+2+1] carbocyclization reaction involving the union of an alkyne, alkene, and carbon monoxide (Scheme 6).^13–15,23^

### 2.1 Diastereoselective reactions

A rare example of a diastereoselective metal-catalyzed [(2+2)+1] reaction using a 1,6-enyne with a 1,1-disubstituted alkene was reported by Yang and coworkers in the total synthesis of (−)-retigeranic acid A.^14*t*^ This process utilizes a cyclopentene containing 1,6-enyne to forge the angular triquinane using the substituted olefin to directly assemble the challenging vicinal quaternary centers in the tricyclic skeleton.

**Fig. 1:**
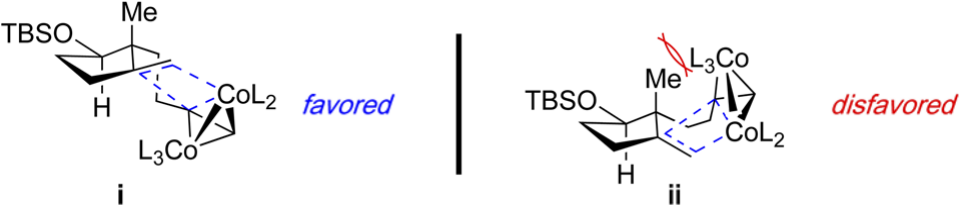
Proposed origin of diastereoselection in the PKR to furnish 2.

Treatment of the 1,6-enyne 1 with stoichiometric Co_2_(CO)_8_ and anhydrous *N*-methylmorpholine *N*-oxide (NMO) as the promoter affords the tricyclic adduct 2 in 72% yield as a single stereoisomer (Table 1, entry 1). Notably, a catalytic reaction variant is possible using tetramethylthiourea (TMTU) with comparable reaction efficiency to the stoichiometric version (entry 1 *vs.* 2). Further improvements were achieved by employing CoBr_2_ as a more stable pre-catalyst in the presence of stoichiometric Zn to generate the active catalytic species (entry 3). The observed diastereoselection is attributed to a steric clash between angular methyl and the alkyne/cobalt cluster in the disfavoured transition state ii (Fig. 1), which affords 2 as a single stereoisomer.

**Table 1:** PKR screening study for the construction of cyclization product 2

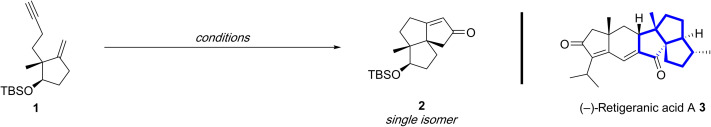		
Entry	Conditions	Yield of 2 (%)
1	Co_2_(CO)_8_ (1.2 equiv.), NMO (4 equiv.), CO (1 atm), toluene, 90 °C	72
2	Co_2_(CO)_8_ (10 mol%), TMTU (60 mol%), CO (1 atm), toluene, 90 °C	55–73
3	CoBr_2_ (10 mol%), TMTU (60 mol%), Zn (2 equiv.), CO (1 atm), 4Å MS, toluene, 90 °C	80

Although it is beyond the scope of this review, an attractive feature of the synthesis is the ability to utilize a second PKR with a 1,7-enyne derived from 2 to directly afford (−)-retigeranic acid A 3 (reaction not shown), albeit with very poor diastereocontrol (*dr* = 2 : 1).

### 2.2 Enantioselective reactions

There are primarily three *traceless* approaches for the asymmetric metal-catalyzed (or metal-mediated) PKR with 1,6-enynes,^24^ namely:

(1) Catalyst/ligand control through the modification of the metal complex with a chiral ligand.

(2) Substrate control that uses a removable chiral auxiliary in the 1,6-enyne; and,

(3) Employing a chiral reagent for asymmetric induction, *e.g.*, a chiral *N*-oxide in the PKR.

A detailed discussion on the latter two is omitted as they generally pertain to metal-mediated processes. Hence, the catalytic enantioselective metal-catalyzed process will be the focus of the following discussion.

The development of enantioselective PKR with 1,6-enynes containing 1,1-disubstituted alkenes follows the chronological order of early-transition metals, *e.g.*, titanium, followed by late-transition metal complexes, such as cobalt, rhodium, and iridium, that now dominate the field. Most of the processes in the review use carbon monoxide gas as a carbonyl source (Section 2.2.1), albeit processes that employ aldehydes as an alternative carbonyl source are also discussed (Section 2.2.2). It is interesting to note that only one substrate with an early-transition metal was examined. In contrast, the late-transition metals have a much broader substrate scope, particularly for rhodium-catalyzed reactions.

#### 2.2.1 PKR using carbon monoxide gas as carbonyl source

##### 2.2.1.1 Ti-catalyzed PKR

**Scheme 7:**
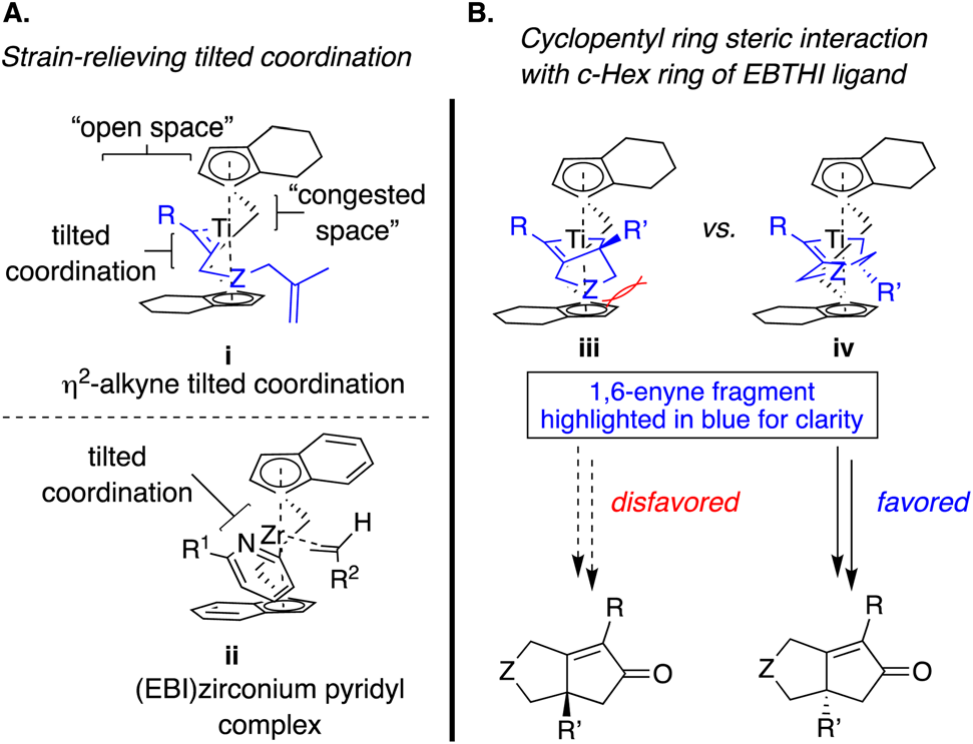
Tilted coordination of alkynyl or pyridyl ligand in the early-transition metal center (left); the proposed enantiodetermining transition state structures in the enantioselective Ti(ii)-catalyzed PKR (right).

Although the original stoichiometric PKR was discovered with a late transition metal, namely, a cobalt complex,^25^ the first example of a highly enantioselective catalytic PKR was reported by Buchwald and Hicks using a chiral titanium catalyst.^15*a*,*b*^ Nevertheless, since this report, no further developments have been made in the use of early transition metals. This study includes only one example of a 1,6-enyne with a 1,1-disubstituted alkene (Table 2, entry 1), which contrasts the terminal alkene derivative that afforded significantly higher enantioselectivity (entry 1 *vs.* 2). Hence, this work provides critical insight into the challenges associated with using substituted alkenes in an enantioselective PKR. The lower enantiocontrol for the 1,1-disubstituted alkene variant is rationalized through the enantiodetermining transition state model shown in Scheme 7.

**Table 2:** The impact of the alkenyl substituent in the 1,6-enyne for the Ti(ii)-catalyzed PKR

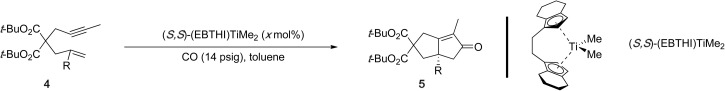				
Entry	R	*x*	Yield (%)	*ee* (%)
1	Me	20	90	72
2	H	5	88	89

The initial *η*^2^-coordination of the Ti(ii)-complex with the alkyne in the 1,6-enyne induces a tilted conformation in i (Scheme 7A, – upper left), which is proposed by analogy with an X-ray crystallographic study with (EBI)zirconium pyridyl complex, where the pyridyl group bound to the zirconium metal center ii.^26^ As a result, this coordination mode predisposes the alkene of the 1,6-enyne to coordinate with the titanium metal center in such a way that the enyne tether backbone minimizes steric repulsion with the nearest bicyclic cyclopentadienyl ring (Cp′ – vi*vs.*iii). Consequently, the coordination positions the prochiral alkenyl substituent (R′) in a pseudoaxial position proximal to the nearest Cp′ (Scheme 7 – right, favored pathway). However, a larger alkenyl substituent (R′) is more likely to destabilize the favored transition state because of the steric impact with the nearest Cp′ (iv), resulting in a smaller difference in the enantiodetermining barriers (ΔΔ*G*^‡^). Therefore, sterically encumbered alkenyl substituents (R′ ≠ H) are expected to result in lower enantiocontrol (Table 2).

##### 2.2.1.2 Co-catalyzed PKR

**Fig. 2:**
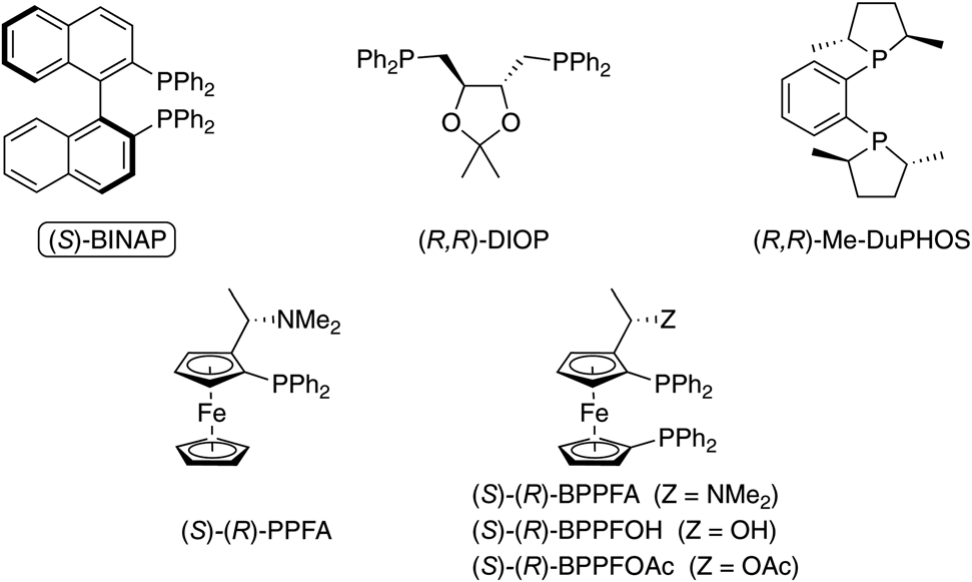
Representative chiral phosphine ligands screened by Hiroi group in the Co-catalyzed PKR.^15*d*,*e*^

In contrast to the enantioselective PKR with early transition metal catalysts (*e.g.*, Ti), late transition metal catalysts (*e.g.*, Co, Rh, and Ir) have been extensively examined since the initial report in 2000. The first enantioselective catalytic PKR involving a late-transition metal complex was reported by Hiroi and coworkers, using a chiral cobalt-complex prepared *in situ* by combining Co_2_(CO)_8_ with a chiral bisphosphine ligand.^15*d*,*e*^ Among the chiral phosphine ligands screened, the enantioenriched 2,2′-bis(diphenylphosphino)-1,1′-binaphthyl (BINAP) is the optimal chiral ligand (Fig. 2).

This observation is instructive as it inspired further studies using similar BINAP-type chiral scaffolds. The popularity of these *C*_2_ atropisomeric chiral ligands is likely due to the availability of variants with systematic variations in the aryl and phosphorus atom substituents. For instance, switching from (*S*)-BINAP to (*S*)-Tol-BINAP probes the impact of the steric difference of the phosphorus atom on the reaction’s reactivity and stereoselectivity. As a result, this enables a systematic examination of ligand stereoelectronic properties during the optimization of a carbocyclization reaction.

This study also highlighted a significant difference in reactivity between terminal and 1,1-disubstituted alkenes with a series of tethered 1,6-enynes (Table 3, entries 1 and 3 *vs.* 2 and 4). The reaction is particularly sensitive to the alkynyl substituent (R^1^ = Me *vs.* H) as switching from a terminal alkyne to a methyl-substituted derivative improved efficiency but resulted in a racemic product (entry 4 *vs.* 5).

**Table 3:** Representative examples of enantioselective Co-catalyzed PKR reported by Hiroi^15*d*,*e*^

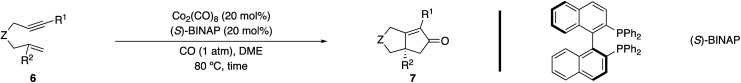						
Entry	Z	R^1^	R^2^	Time (h)	Yield (%)	*ee* (%)
1	NTs	H	H	15	60	93
2	"	"	Me	14	13	62
3	C(CO_2_Me)_2_	"	H	"	53	90
4	"	"	Me	24	31	63
5	"	Me	"	"	90	0

##### 2.2.1.3 Ir-catalyzed PKR

Shibata and coworkers concurrently reported the first examples of enantioselective Ir(i)-catalyzed PKR with 1,6-enynes using a chiral complex derived from [Ir(COD)Cl]_2_ and (*S*)-Tol-BINAP under a CO atmosphere (1 atm) in refluxing toluene.^15*g*^ Preliminary studies with a 1,6-enyne containing a 1,1-disubstituted alkene were inefficient and afforded modest enantioselectivity (Table 4, entry 1). Increasing the reaction temperature by switching to refluxing xylene improved the yield but slightly reduced enantioselectivity (entry 1 *vs.* 2). Additional studies explored the effect of lowering the CO partial concentration, which significantly improved the efficiency and enantioselectivity (entries 1 *vs.* 3 and 4 *vs.* 5).^15*o*^ These findings are consistent with the work of Schmid and Consiglio using a cationic Rh(i) catalyst with 1,6-enynes that have a terminal alkene (not shown).^15*l*^ However, the examples are limited to oxygen-tethered substrates with an alkynyl phenyl substituent (*e.g.*, 1,6-enyne 8), which is the most common substrate examined in related enantioselective metal-catalyzed PKR with 1,1-disubstituted alkenes.

**Table 4:** The impact of carbon monoxide partial pressure on the enantioselective Ir(i)-catalyzed PKR with 1,6-enynes containing various alkenyl substituents

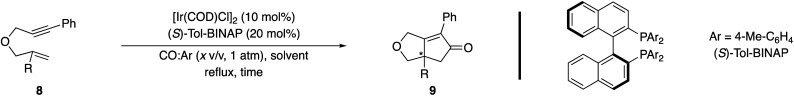						
Entry	R	Solvent	CO : Ar (*x* v/v)	Time (h)	Yield (%)	*ee* (%)
1	Me	Toluene	100 : 0	24	30	88
2	"	Xylene	"	"	51	82
3	"	Toluene	20 : 80	72	86	93
4	Allyl	"	100 : 0	96	22	86
5	"	"	20 : 80	"	62	94

**Scheme 8:**
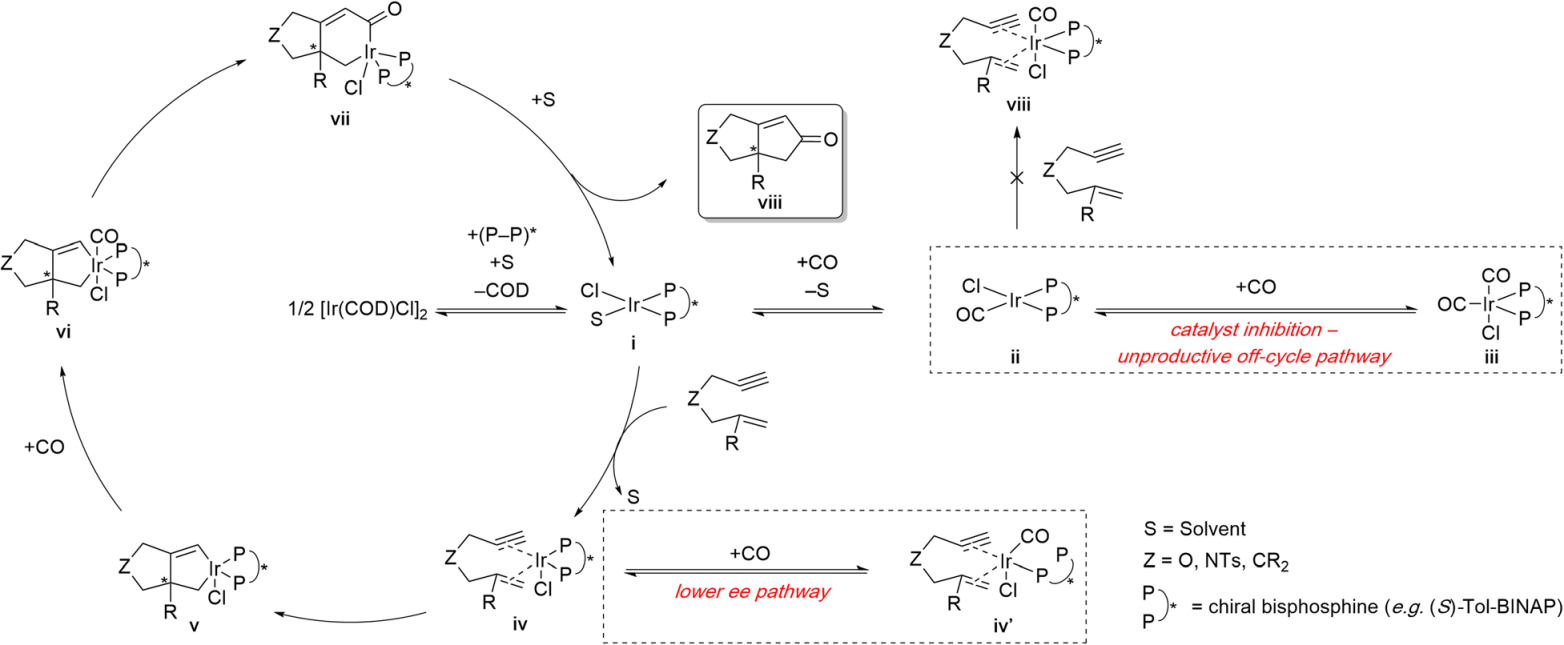
Proposed mechanism for the neutral Ir(i)-catalyzed enantioselective PKR.

Shibata and coworkers proposed a mechanism for the enantioselective Ir(i)-catalyzed PKR that accounts for the impact of CO partial pressure (Scheme 8).^15*g*,*o*^ The catalytically active complex i forms through ligand dissociation of a cycloocta-1,5-diene (COD) ligand, followed by association of the chiral bisphosphine, and stabilized by a solvent molecule. Complex i can either enter the productive PKR cycle by coordinating the 1,6-enyne or participate in the unproductive off-cycle pathway (Scheme 8 – right center, dashed box). The latter pathway involves successive coordination with carbon monoxide, forming the catalytically inactive metal complexes ii and iii, which are unable to fully coordinate the 1,6-enyne. The mechanism accounts for the improved PK reactivity at lower CO partial pressure (entries 1 *vs.* 3 and 4 *vs.* 5). The productive PKR pathway *via* intermediate iv undergoes rate- and enantiodetermining oxidative addition to form metallacycle intermediate v. However, intermediate iv can also undergo partial decomplexation of one of the phosphines from the chiral bisphosphine ligand to free up a vacant coordination site. Coordination of CO to the vacant coordination site forms intermediate iv′ that can also undergo carbocyclization, albeit the level of enantiocontrol would likely be poor because the chiral bisphosphine is only bound through one of the phosphines (Scheme 8 – lower right, dashed box). This mechanism also explains the improved enantioselectivity observed at lower CO partial pressure (*cf.* entries 1 *vs.* 3 and 4 *vs.* 5). To complete the catalytic cycle, CO coordinates with intermediate v, inserts into Ir–C(sp^2^) bond, and forms the acyl intermediate vii, albeit the alternative M–C(sp^3^) has also been proposed.^27^ Reductive elimination of intermediate vii and subsequent catalyst decomplexation from product viii regenerates the active catalyst i.

Pfaltz and coworkers demonstrated that a chiral cationic Ir(i)-complex^15*s*^ also facilitates an enantioselective PKR. The chiral complex was derived from a chiral phosphinooxazoline (PHOX) type ligand, a ligand commonly employed in enantioselective metal-catalyzed allylic alkylations.^28^ Notably, 1,6-enynes with 1,1-disubstituted alkenes proved to be poor substrates compared to their terminal alkene counterparts (Table 5, entries 1 and 2 *vs.* 3 and 4). Interestingly, the complex with a triflate (TfO^−^) counter-anion afforded higher enantiocontrol compared to the larger and less coordinative counterpart (BAr_*F*_^−^: tetrakis(3,5-bis(trifluoromethyl)phenyl)borate). Nevertheless, no clear correlation is observed between reaction efficiency and the choice of counter-anion (entries 1 and 2 *vs.* 3 and 4 *vs.* 5 and 6).

**Table 5:** Effect of the alkenyl substituent and counteranion in a cationic enantioselective Ir(i)-catalyzed PKR

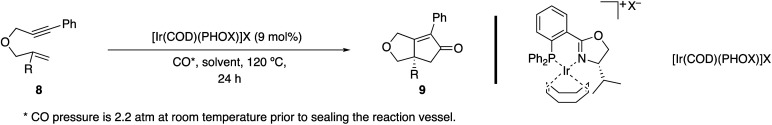					
Entry	R	X	Solvent	Yield (%)	*ee* (%)
1	H	TfO^−^	DME	85	91
2	"	BAr_*F*_^−^	"	69	85
3	Me	TfO^−^	"	9	58
4	"	BAr_*F*_^−^	"	Trace	—
5	"	TfO^−^	THF	10	71
6	"	BAr_*F*_^−^	"	28	64

##### 2.2.1.4 Rh-catalyzed PKR

**Scheme 9:**
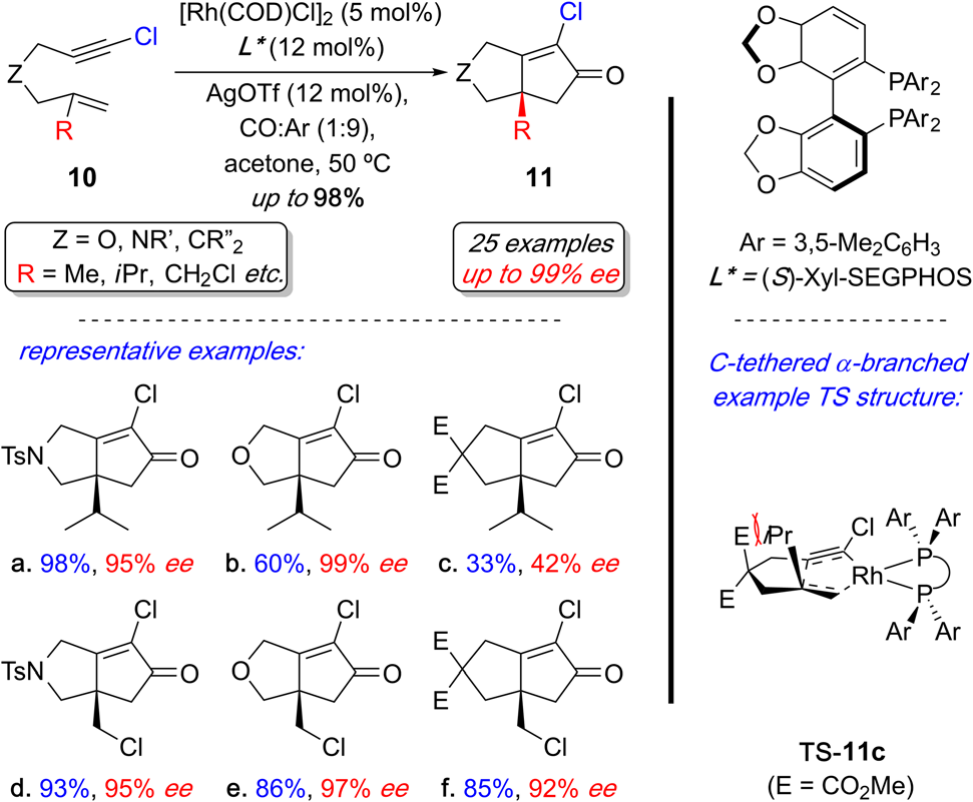
Overview of Rh(i)-catalyzed enantioselective PKR with 1,6-choroenynes with representative examples.

In 2023, Evans and coworkers developed a Rh(i)-catalyzed enantioselective PKR with 1,6-chloroenynes.^15*ae*^ They demonstrated that 1,6-chloroenynes 10 enable a mild, enantioselective process to prepare a broad range of PK products 11 with quaternary stereogenic centers, typically achieving excellent yields and enantioselectivity (Scheme 9). Although, sterically challenging α-branched examples are tether dependent (*cf*. 11a–c), the labile allylic chlorides are remarkably tolerant and unaffected by the tether (*cf.*11d–f). The origin of the poor reactivity and selectivity with the carbon-tethered α-branch example (11c) was gleaned using DFT calculations—the enantiodetermining transition state (TS) structure that forms 11c implicates an undesired steric clash between one of the ester groups with the α-branch substituent during the key metallacycle formation step (TS-11c). In contrast, the nitrogen- and oxygen-tethered counterparts do not have this detrimental steric interaction. This process provides access to the first examples of challenging PK products with quaternary stereogenic centers using relatively mild reaction conditions.

The report also delineated the stereoelectronic influence exerted by the alkynyl chloride in the enyne on the reactivity and enantioselectivity in this reaction by comparing DFT computational models with experimental results. The alkynyl chloro substituent plays a dual-function during the metallacycle formation step (rate- and enantiodetermining step): (i) *Electronic*: the chloro-substituent pre-polarizes the alkyne to lower the barrier for metallacycle formation; and (ii) *Steric*: the relatively small chloro substituent permits a tighter substrate (1,6-chloroenyne)–ligand interaction thereby enhancing enantioselectivity through a stronger enantiodetermining interaction.

#### 2.2.2 PKR using aldehydes as carbonyl source

The enantioselective metal-catalyzed PKRs discussed so far (Ti, Co, Ir, and Rh) directly utilize CO gas as a reagent. In contrast, only the Rh(i)- and Ir(i)-catalyzed variants with 1,6-enynes containing 1,1-disubstituted olefins use alternative CO sources, such as cinnamaldehyde and other aldehydes. However, the studies in this section are limited to a single substrate, the 1,6-enyne 8, because it has the optimal tether and alkynyl substituent to facilitate an efficient and selective reaction. This limitation significantly reduces the broader synthetic applicability of the transformation.

**Scheme 10:**
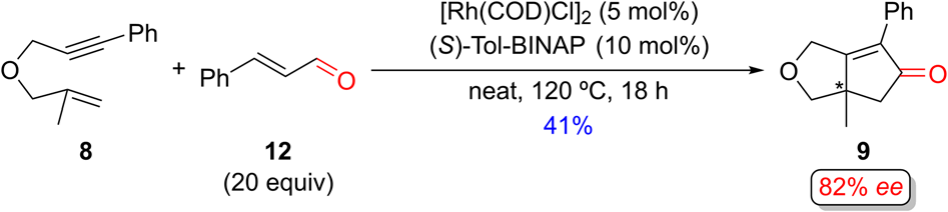
An example of a Rh(i)-catalyzed enantioselective PKR of a 1,6-enyne with cinnamaldehyde as an alternative CO source by Shibata and coworkers.^15*h*^

Several groups have independently reported using aldehydes as an alternative CO source in racemic PKRs.^29^ Shibata and coworkers reported the first enantioselective Rh(i)-catalyzed PKR of the 1,6-enyne 8 using cinnamaldehyde as the CO source to afford PK adduct 9 in 41% yield and with 82% *ee* (Scheme 10).^15*h*^ Interestingly, they also examined using [Ir(COD)Cl]_2_/(*S*)-Tol-BINAP catalyst system with cinnamaldehyde (5 equiv.) under similar conditions, which produced the PK product 9 in 40% yield with improved enantiomeric excess (90%).^15*o*^ Additionally, Chan and coworkers achieved excellent enantioselectivity (96% *ee*) for product 9 using [Ir(COD)Cl]_2_/(S)-BINAP as the catalyst and nonylaldehyde as the carbonyl source in dioxane (not shown).^15*q*^

**Scheme 11:**
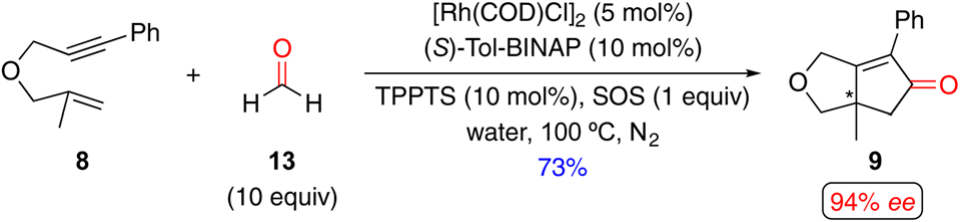
An example of a Rh(i)-catalyzed enantioselective PKR with 1,6-enyne and formaldehyde as an alternative CO source by Morimoto and coworkers.^15*m*^

Enantioselective Rh(i)-catalyzed PKRs with CO equivalents can also be performed in protic reaction solvents like alcohol and water,^15*m*,30^ which are considered “greener” alternatives.^31^ For example, Morimoto and coworkers reported an enantioselective Rh(i)-catalyzed PKR of 1,6-enyne 8 with formaldehyde 13 as a CO equivalent, along with sodium octadecylsulfate (SOS) as a surfactant and triphenylphosphine-3,3′,3′′-trisulfonic acid trisodium salt (TPPTS) as the hydrophilic phosphine, to furnish the PK product 9 in 73% yield and with 94% *ee* (Scheme 11).^15*m*^

**Scheme 12:**
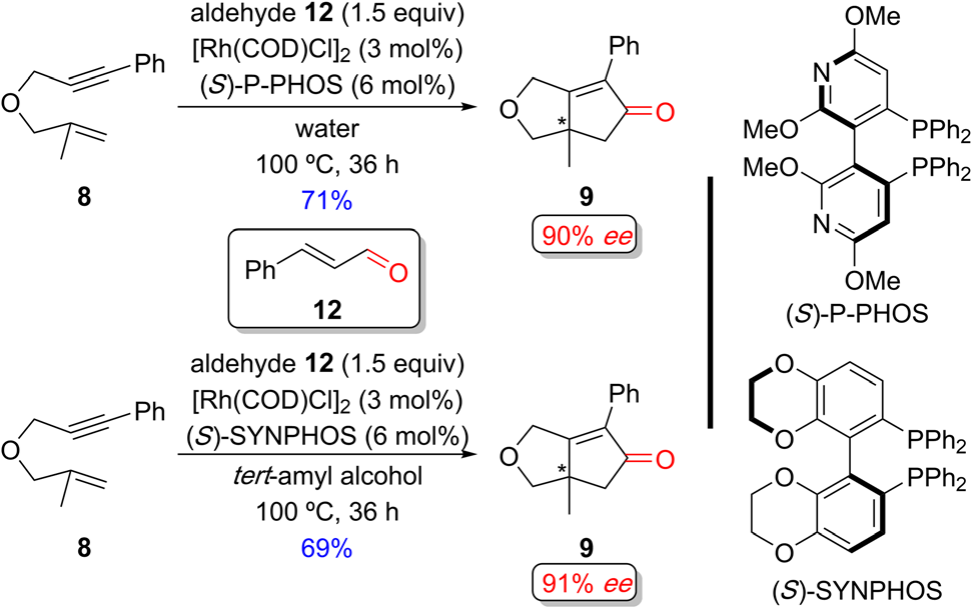
Examples of a Rh(i)-catalyzed enantioselective PKR of a 1,6-enyne and cinnamaldehyde as an alternative CO source without surfactants by Chan and coworkers.^15*n*,*p*^

Chan and coworkers developed an enantioselective PKR with aldehydes in protic solvents without a surfactant (Scheme 12).^15*n*,*p*^ Previous studies indicated that switching from an organic solvent (such as toluene, dioxane, DMF, *etc.*) to water significantly improved the yield and enantioselectivity for the PKR adduct (not shown). The origin of the improved efficiency and selectivity is ascribed to the aqueous conditions increasing the “effective concentration” of reactants, a concept similar to the effect observed in the aqueous micellar system.^32^

**Scheme 13:**
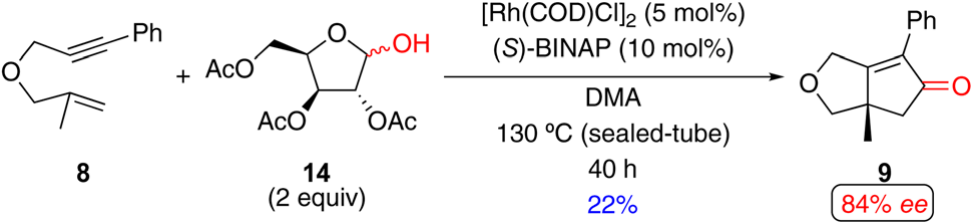
An example of a Rh(i)-catalyzed enantioselective PKR of a 1,6-enyne with aldose derivatives as an alternative CO source by Ikeda and coworkers.^15*ab*^

Ikeda and coworkers utilized aldose and its derivatives as an alternative CO source for an enantioselective Rh(i)-catalyzed PKR (Scheme 13),^15*ab*^ a concept similar to the racemic examples reported by Chung.^33^ In this reaction, 1,6-enyne 8 with acetyl-protected d-xylose 14 affords the PK product in 22% yield with 84% *ee* and considerable quantities of the unreacted 1,6-enyne 8 (47%). Ikeda's approach is constrained by the requirement for high temperature (130 °C) and the use of acetylated aldose (a CO source that requires extra synthetic steps to prepare).

Overall, the current state of the enantioselective metal-catalyzed PKR of 1,6-enynes with 1,1-disubstituted alkenes is limited by the choice of tethering atom and alkyne substituent. Additionally, the scope of alkenyl substituents is primarily limited to methyl and allyl groups, particularly for achieving high yield and enantioselectivity. These limitations present a significant challenge, necessitating a more general solution to broaden the scope of this synthetically useful transformation in target-oriented synthesis. In this regard, the recent introduction and use of 1,6-chloroenynes offers a promising approach that can be applied to related carbocyclizations.

## 3. Metal-catalyzed [(2+2)+2] carbocyclization reactions

The stereoselective metal-catalyzed [(2+2)+2] carbocyclization reaction of 1,6-enynes provides a critical and highly convergent approach for constructing [5,6]-bicyclic scaffolds that are present in bioactive agents. Although 1,6-enynes with terminal alkenes are frequently studied, derivatives with a 1,1-disubstituted alkene, including tri- and tetrasubstituted alkenes, are comparatively rare or unknown.^16–18,34,35^ The following section delineates the stereoselective metal-catalyzed [(2+2)+2] reactions of 1,6-enynes with 1,1-disubstituted alkenes, using exogenous alkynes and alkenes to illustrate the limited substrate scope.

### 3.1 Diastereoselective reactions

**Scheme 14:**
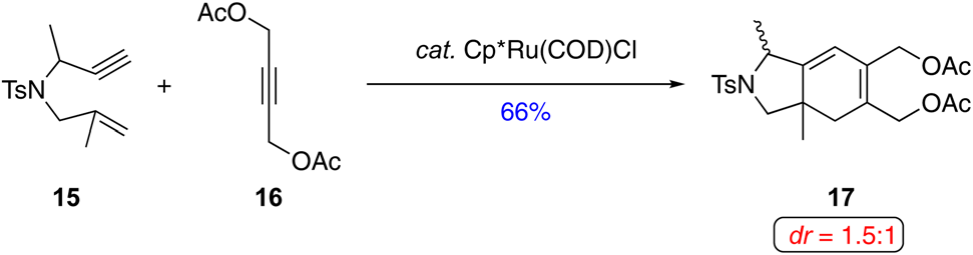
An example of a diastereoselective Ru(ii)-catalyzed [(2+2)+2] of a 1,6-enyne with an exogenous alkyne by Tenaglia and coworkers.^36^

One of the few reported examples of a diastereoselective metal-catalyzed [(2+2)+2] carbocyclization involving a 1,6-enyne with a 1,1-disubstituted alkene and an exogenous alkyne highlights the potential for further advancements in this area. In 2017, Tenaglia and coworkers reported a diastereoselective ruthenium-catalyzed [(2+2)+2] carbocyclization of 1,6-enyne and symmetrical alkyne (Scheme 14).^36^ Treatment of 1,6-enyne 15 and alkyne 16 with Cp*Ru(COD)Cl afforded cyclized product 17 in moderate yield and poor diastereoselectivity (*dr* = 1.5 : 1), underscoring the current challenges with this approach.

### 3.2 Regio- and enantioselective reactions

#### 3.2.1 Rh-catalyzed [(2+2)+2] of 1,6-enynes and exogenous alkynes

**Scheme 15:**
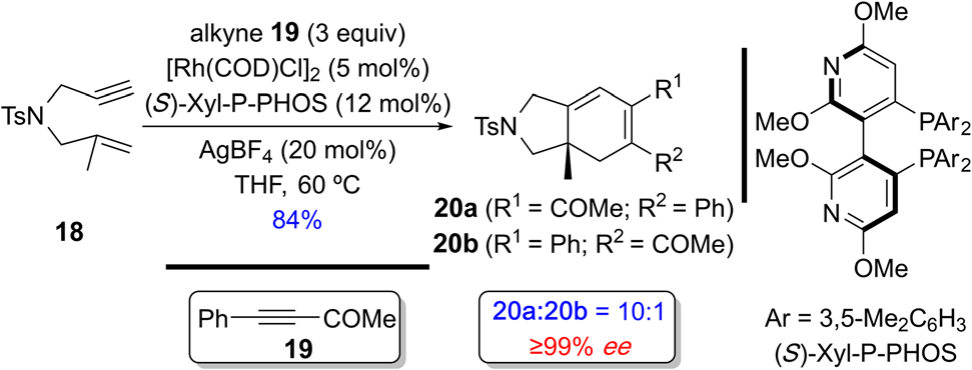
An example of a regio- and enantioselective Rh(i)-catalyzed [(2+2)+2] of a 1,6-enyne with an exogenous alkyne by Evans and coworkers.^18*a*^

In 2005, Evans and Shibata independently developed the enantioselective Rh(i)-catalyzed [(2+2)+2] carbocyclization of 1,6-enynes with alkynes to afford the bicyclohexadienes with a quaternary center.^18*a*,*e*^ Consequently, the former described the reaction of 1,6-enyne 18 with alkyne 19 using the cationic chiral Rh(i) catalyst generated from [Rh(COD)Cl]_2_, (*S*)-Xyl-P-PHOS and AgBF_4_ to afford the cyclized products 20a/b in 84% yield with good regio- and excellent enantioselectivity favoring 20a (Scheme 15).^18*a*^

**Scheme 16:**
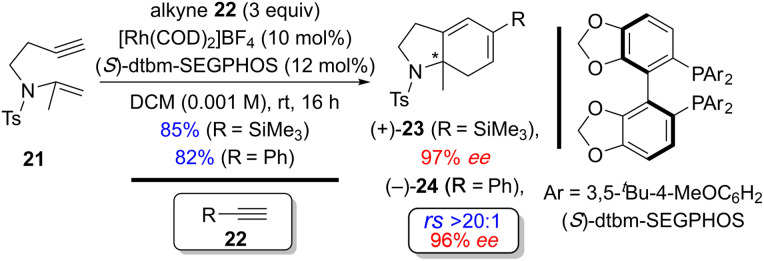
Examples of regio- and enantioselective Rh(i)-catalyzed [(2+2)+2] of homopropargyl enamides (tosylamide-tethered 1,6-enyne) with exogenous alkynes by Tanaka and coworkers.^18*d*^

Recently, Tanaka and coworkers demonstrated the regio- and enantioselective Rh(i)-catalyzed [(2+2)+2] carbocyclization reaction of the homopropargyl enamides (tosylamide-tethered 1,6-enynes) with alkynes to construct tetrahydroindole skeletons at room temperature. Interestingly, the concentration of substrate 21, which contains a 1,1-disubstituted alkene, is critical for garnering good efficiency and selectivity. For example, the efficiency and selectivity for the formation of (+)-23 improved significantly from 65% yield and 60% *ee* at high concentration (0.02 M) to 85% yield and 97% *ee* at low concentration (0.001 M) (Scheme 16).^18*d*^

### 3.3 Regio-, diastereo-, and enantioselective reactions

Although extending the exogenous component to alkenes can introduce up to two stereogenic centers in the product, controlling regio-, diastereo-, and enantioselectivity is challenging. Consequently, the examples described in this section require a Lewis basic atom in the exogenous alkene to ensure a productive cyclization process, potentially limiting the scope of this process.

#### 3.3.1 Monosubstituted alkenes as exogenous component

In 2012, Tanaka and coworkers reported the first example of a regio-, diastereo-, and enantioselective Rh(i)-catalyzed [(2+2)+2] carbocyclization reaction of 1,6-enynes with acrylamides as the exogenous component.^18*f*^ The substituents in the 1,1-disubstituted alkene component of the 1,6-enyne are primarily limited to methyl, ethyl, and one example of a phenyl group. Notably, the yields and enantioselectivities generally decrease as the size of the alkene substituent increases (Table 6, entries 1–3), which is tentatively ascribed to an increase in the barrier for metallacycle formation. Furthermore, all examples with sulfonamide- and malonate-tethered 1,6-enynes proceed with moderate to excellent yields and enantioselectivities. While there is only one example of an oxygen-tether, it furnished the product with moderate yield and excellent enantioselectivity (not shown).

**Table 6:** Representative examples of a regio- and stereoselective Rh(i)-catalyzed [(2+2)+2] of 1,6-enynes containing various alkynyl and alkenyl substituent with an exogenous acrylamide

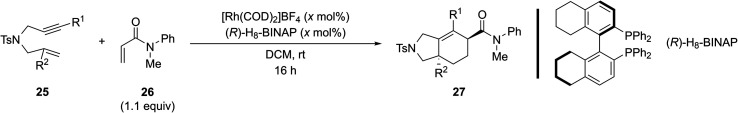					
Entry	*x*	R^1^	R^2^	Yield (%)	*ee* (%)
1	3	Me	Me	>99	>99
2	5	"	Et	86	>99
3	10	H	Ph	47	94

**Scheme 17:**
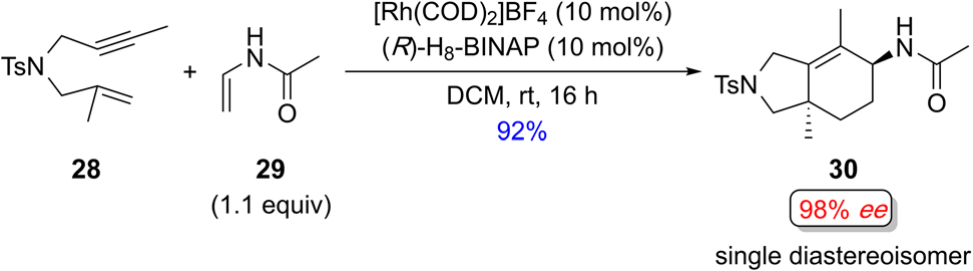
Representative example of a diastereo- and enantioselective Rh(i)-catalyzed [(2+2)+2] of a 1,6-enyne with an exogenous enamide.

In 2016, the same group reported a similar regio- and stereoselective reaction using enamides instead of acrylamides (Scheme 17).^18*g*^ All the examples afforded the cyclized products with excellent regio-, diastereo-, and enantiocontrol for the alkene insertion product, which leads to the creation of two non-adjacent stereogenic centers. However, only sulfonamide- and malonate-tethered 1,6-enynes were examined.

The studies conducted by Tanaka and coworkers with acrylamides^18*f*^ and enamides^18*g*^ indicate that the relative electron density of the exogenous alkene component does not significantly impact reaction efficiency and stereocontrol. Notably, a Lewis basic substituent is necessary to facilitate coordination with the metallacycle to ensure an efficient [(2+2)+2] reaction. This hypothesis is indirectly supported by the observation that exogenous alkenes without a coordinating atom are not reported.

In 2020, Tanaka and coworkers reported the kinetic resolution of racemic secondary allylic alcohols (5 equiv.) in the [(2+2)+2] carbocyclization with 1,6-enynes (Table 7).^18*i*^ This reaction produced the carbocyclization product with three stereogenic centers in a one step, affording a single diastereoisomer with excellent enantiocontrol. Although the scope included four examples of 1,6-enynes with 1,1-disubstituted alkenes, oxygen-tethered derivatives were excluded from this study.

**Table 7:** Representative examples of the kinetic resolution of racemic secondary allylic alcohols in a Rh(i)-catalyzed [(2+2)+2] carbocyclization reaction

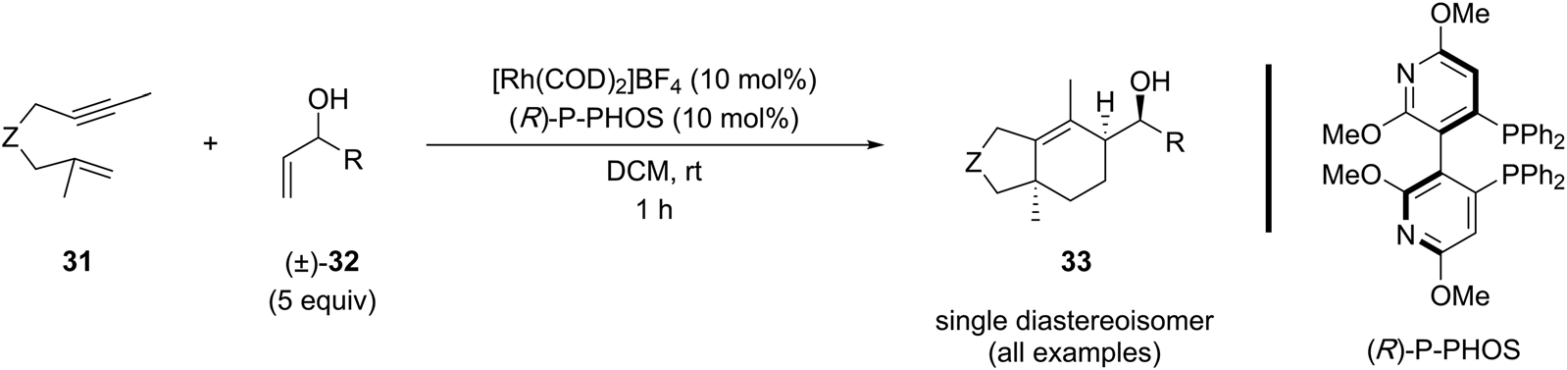				
Entry	Z	R	Yield (%)	*ee* (%)
1	NTs	Ph	68	99
2	"	*c*-Hex	79	99
3	"	*t*-Bu	20	>99
4	C(CO_2_Bn)_2_	*c*-Hex	42	99

The study also highlights the importance of the unprotected hydroxyl group in the allylic alcohol component for a successful [(2+2)+2] reaction, since experiments using methylated allylic alcohol and homoallylic alcohol derivatives confirmed that these compounds are unreactive (Table 8, entries 1 *vs.* 2 and 3). These findings emphasize the importance of the hydroxyl group and its optimal distance for coordinating the metal center, which provides valuable insight into the reaction mechanism.

**Table 8:** Control experiments for the regio- and stereoselective Rh(i)-catalyzed [(2+2)+2] carbocyclization reaction with 1,6-enynes with racemic allylic alcohols

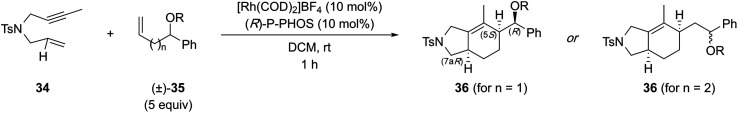				
Entry	R	*n*	Yield (%)	*ee* (%)
1^a^	H	1	60	98
2	Me	1	0	—
3	H	2	0	—

a *dr* = (*R*, 5*S*, 7a*R*) : (*S*, 5*R*, 7a*R*) : {two unspecified diastereoisomer} = 7 : 2.5 : 1.

#### 3.3.2 1,2-Disubstituted alkenes as exogenous component

In 2019, Zhou and Fan extended the Rh(i)-catalyzed [(2+2)+2] carbocyclization to oxabenzonorbornadienes as an exogenous 1,2-disubstituted alkenes (Table 9).^18*j*^ Notably, only the methyl-substituted 1,6-enyne with carbon and heteroatom tethers were examined, highlighting the limitations of this approach. For instance, the sulfonamide-tethered derivative provides varied results that are a direct consequence of the size of the alkynyl substituent (entries 1–3), whereas the terminal alkyne is significantly more efficient and selective than the substituted derivatives. In contrast, the oxygen- and malonate-tethered enynes are either inefficient or unreactive, even with the unsubstituted alkyne, illustrating the challenges with this process (entries 4–5).

**Table 9:** Representative examples of a stereoselective Rh(i)-catalyzed [(2+2)+2] of 1,6-enynes with exogenous 1,2-disubstituted alkenes

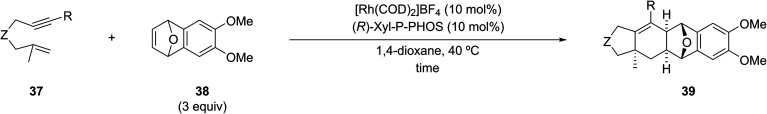					
Entry	Z	R	Time (h)	Yield (%)	*ee* (%)
1	NTs	H	4	58	99
2^a^	"	Me	3	12	81
3	"	Ph	48	NR	—
4	O	H	3	17	87
5	C(CO_2_Me)_2_	"	72	NR	—

a The reaction condition was modified with DCE as solvent at 60 °C instead.

#### 3.3.3 Trisubstituted alkenes as exogenous component

In 2016, Tanaka and coworkers reported a regio- and stereoselective Rh(i)-catalyzed [(2+2)+2] carbocyclization of 1,6-enynes with a 1,1-disubstituted alkene and an exogenous trisubstituted alkene, specifically cyclopropylideneacetamides.^18*h*^ This study required a higher loading of Rh(i)-catalyst (15–20 mol%) compared to their previous work with terminal alkenes, where only 3–10 mol% of the catalyst is typically used.^18*f*,*g*,*i*^ Nitrogen-tethered substrates proceed with moderate to good yields and excellent enantioselectivities, albeit switching to a terminal alkyne resulted in poor yield (Table 10, entry 1 *vs.* 4). Additionally, changing the alkenyl substituent from methyl to ethyl resulted in lower yield and slightly reduced enantiocontrol (entry 1 *vs.* 2), indicating that the alkenyl substituents affect the reaction scope in the 1,6-enyne. Oxygen- and carbon-tethered substrates also presented challenges (entry 1 *vs.* 5 and 6), a trend observed in related studies by the same group^18*f*,*g*,*i*^ and others.^18*j*^

**Table 10:** Representative examples of a regio- and stereoselective Rh(i)-catalyzed [(2+2)+2] of 1,6-enynes with exogenous trisubstituted alkenes

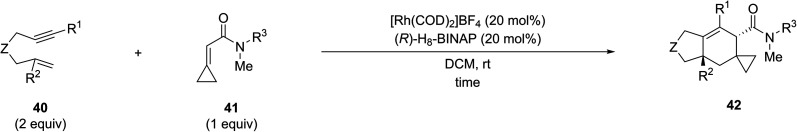							
Entry	Z	R^1^	R^2^	R^3^	Time (h)	Yield (%)	*ee* (%)
1	NTs	Me	Me	Ph	24	69	>99
2	"	"	Et	"	"	51	97
3	"	"	Me	Me	"	75	>99
4	"	H	"	"	"	21	98
5	O	*n*-C_5_H_11_	"	Ph	48	0	—
6	C(CO_2_Me)_2_	Me	"	"	"	<5	—

## 4. Conclusions

Overall, the scope of 1,6-enynes containing a 1,1-disubstituted alkene that can undergo stereoselective metal-catalyzed [(2+2)+1] and [(2+2)+2] carbocyclization reactions is limited. This restricted scope arises from the narrow range of tether atoms and substituents on the alkyne and alkene that are compatible with both reactivity and stereoselectivity. Although strategies to enhance these features have been developed, they predominantly rely on 1,6-enynes containing terminal alkenes rather than the significantly less reactive 1,1-disubstituted variants. Consequently, the full potential of the latter in the total synthesis of complex natural products has yet to be fully realized. Nevertheless the stereoselective carbocyclization reactions that form a quaternary stereogenic center from a prochiral 1,6-enyne in a single operation, makes it an attractive strategy for rapidly constructing challenging polycyclic scaffolds present in valuable synthetic intermediates.

The recent emergence of 1,6-chloroenynes offers a promising solution to the limitations with challenging carbocyclizations, as exemplified by their success in the Pauson–Khand reaction. This potential could also be extended to other related carbocyclizations involving 1,6-enynes. Hence, the 1,6-chloroenyne shows great promise for expanding the traditionally restricted scope in various carbocyclizations, thereby offering new possibilities for discovering novel reactivities that have remained untapped due to prohibitive reaction barriers under conventional conditions.

## Data availability

No primary research results, software, nor code have been included and no new data were generated or analyzed as part of this review.

## Author contributions

Ridge Michael P. Ylagan initiated, drafted, and proofread the manuscript. Yu Zhu revised the document by adding sections and references, and conducted extensive proofreading. P. Andrew Evans edited and proofread the manuscript.

## Conflicts of interest

There are no conflicts to declare.
